# A Deep Learning-Based Watershed Feature Fusion Approach for Tunnel Crack Segmentation in Complex Backgrounds

**DOI:** 10.3390/ma18010142

**Published:** 2025-01-01

**Authors:** Haozheng Wang, Qiang Wang, Weikang Zhang, Junli Zhai, Dongyang Yuan, Junhao Tong, Xiongyao Xie, Biao Zhou, Hao Tian

**Affiliations:** 1 Zhejiang Scientific Research Institute of Transport, Hangzhou 311305, China; whzwy007@163.com (H.W.); wlzhang2010@gmail.com (W.Z.); wwwzhaijunli9@163.com (J.Z.); yuan_dongyang@163.com (D.Y.); zjhzzjutjh@163.com (J.T.); tianhao_8@163.com (H.T.); 2 Key Laboratory of Road and Bridge Detection and Maintenance Technology of Zhejiang Province, Hangzhou 311305, China; 3 Department of Geotechnical Engineering, College of Civil Engineering, Tongji University, Shanghai 200092, China; xiexiongyao@tongji.edu.cn (X.X.); zhoubiao@tongji.edu.cn (B.Z.); 4 Key Laboratory of Geotechnical and Underground Engineering, Ministry of Education, Tongji University, Shanghai 200092, China

**Keywords:** tunnel detection, machine vision, deep learning, lining cracks, image recognition

## Abstract

As highway tunnel operations continue over time, structural defects, particularly cracks, have been observed to increase annually. Coupled with the rapid expansion of tunnel networks, traditional manual inspection methods have proven inadequate to meet current demands. In recent years, machine vision and deep learning technologies have gained significant attention in civil engineering for the detection and analysis of structural defects. However, rapid and accurate defect identification in highway tunnels presents challenges due to complex background conditions, numerous interfering factors, and the relatively low proportion of cracks within the structure. Additionally, the intensive labor requirements and limited efficiency in labeling training datasets for deep learning pose significant constraints on the deployment of intelligent crack segmentation algorithms. To address these limitations, this study proposes an automatic labeling and optimization algorithm for crack sample sets, utilizing crack features and the watershed algorithm to enable efficient automated segmentation with minimal human input. Furthermore, the deep learning-based crack segmentation network was optimized through comparative analysis of various network depths and residual structure configurations to achieve the best possible model performance. Enhanced accuracy was attained by incorporating axis extraction and watershed filling algorithms to refine segmentation outcomes. Under diverse lining surface conditions and multiple interference factors, the proposed approach achieved a crack segmentation accuracy of 98.78%, with an Intersection over Union (IoU) of 72.41%, providing a robust solution for crack segmentation in tunnels with complex backgrounds.

## 1. Introduction

Tunnels, as a mode of transportation for crossing mountainous regions, play a significant role in improving highway alignment, reducing travel distances, and enhancing driving comfort [1]. While tunnels effectively serve their intended functions, the increasing number and cumulative mileage of tunnels, along with the extended operational periods, have gradually led to deterioration in their service capabilities [2]. Meanwhile, an increasing number of structural defects have emerged, making the previously manual-based inspection methods inadequate to address current maintenance and inspection demands. Therefore, machine vision-based methods for detecting and analyzing the apparent defects of tunnels have been widely used in recent years [3,4]. However, it is necessary to segment images at the pixel level from the massive image data collected by machine vision, analyze and extract defect parameters based on the segmentation results, and provide data support for conducting detailed evaluations of tunnel service performance. Furthermore, due to variations in highway tunnel structures and management practices, discrepancies exist in decorative layers, electromechanical equipment, and cleanliness. These factors contribute to complex backgrounds in tunnel lining images, which often contain numerous interfering elements such as stains, cobwebs, construction joints, and leakage traces. This poses a significant challenge for the rapid and intelligent identification of tunnel defects. Among the various apparent defects of the tunnel, it is difficult to automatically identify and segment the cracks due to their small area relative to the image and the presence of numerous interference elements with similar shapes to cracks [5]. Consequently, this paper focuses on the segmentation of cracks caused by the cracking of tunnel lining structures.

Currently, general crack segmentation algorithms are mainly divided into two categories: the first involves manually selecting crack features and implementing the corresponding algorithm through programming, known as the feature-based algorithms; the second is to build a crack sample library and then use deep learning algorithms to select features and generate weight parameter models for similar crack recognition and segmentation, referred to as deep learning -based algorithms.

In feature-based crack segmentation algorithms, Roberts [6,7], Prewitt [8,9], Sobel [10,11], Canny [12,13], and Laplacian [9,14] edge gradient detection operators, along with their improved algorithm for cracks [15,16], are used to detect the borders through gray gradient changes. Principles such as infiltration and minor path principles are used to optimize crack segmentation based on neighboring pixels. The OTSU [17] algorithm performs threshold segmentation by determining the optimal threshold between crack pixels and background pixels, with improvements made by filtering and noise reduction techniques [18,19].

In deep learning-based algorithms, GAO Y W [20] combined the FASTER RCNN, ROI boundary adaptation, and a fully convolutional neural network to reduce the error rate of infiltration water recognition to as low as 0.02. Huang Hongwei [21] designed dual FCN models, showing a better segmentation performance on six types of images with cracks, leakage, and their combinations. In addition, Huang Hongwei [22] introduced morphological closing operations to optimize the segmentation results of the Mask R-CNN. In testing across 76 test sets, the model achieved an accuracy of 81.94%, an F1 value of 68.68%, and an IOU of 52.72%.

Feature-based crack segmentation algorithms have poor anti-interference ability, leading to frequent misunderstandings on tunnel lining with more interference such as seepage traces and spider webs, which affects the accuracy of the segmentation. However, due to their reliance on standard image features, these algorithms typically achieve precise edge segmentation for cracks [23,24].

Deep-learning crack segmentation algorithms require extensive manual labeling of crack samples before training. However, relying on subjective judgment to determine gray-scale crack boundaries is often insufficiently precise, especially for narrow cracks with fewer pixels in width. Additionally, due to the limited generalization capacity of deep learning models, the segmentation accuracy of the model decreases significantly when there are changes in the lining coating material and background interference. Furthermore, deep learning segmentation algorithms segment pixels based on the probabilities of different pixel categories. Since the probabilities of background and crack pixels are typically similar at the edges of cracks, this area becomes a primary source of crack segmentation errors.

Therefore, this paper proposes a method that integrates feature segmentation and deep learning segmentation, improving the efficiency of crack labeling and enhancing the crack segmentation effect based on deep learning. In Section 2 of this paper, the image acquisition process is first introduced, and the characteristics of crack images with complex backgrounds are analyzed. In Section 3, an automatic crack segmentation method and an optimization method using the watershed algorithm are established. In Section 4, an algorithm for optimizing the U-NET network model using methods such as adjusting the number of network layers and employing residual structures is proposed. In Section 5, commonly used segmentation accuracy evaluation metrics are introduced, and a comparison of the performance of the U-NET model before and after optimization is conducted, demonstrating the superiority of the algorithm.

## 2. Tunnel Crack Image Acquisition and Analysis

### 2.1. Tunnel Crack Image Acquisition

**Figure 1:**
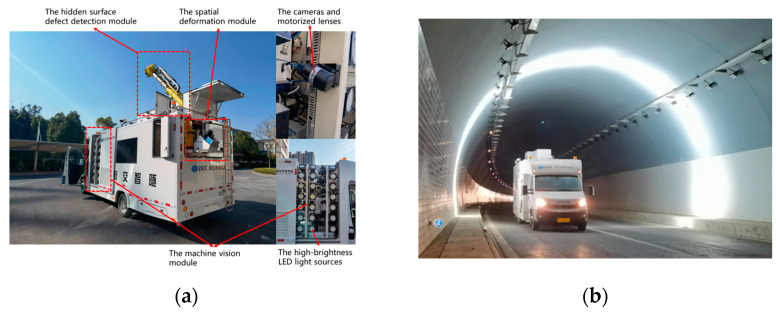
“ZHE JIAO ZHI SUI” intelligent comprehensive tunnel inspection vehicle (ZJZS inspection vehicle): (**a**) Modular composition; (**b**) Field operations.

To efficiently and rapidly collect data on tunnel lining defects within tunnels, the “ZHE JIAO ZHI SUI” intelligent comprehensive tunnel inspection vehicle (ZJZS inspection vehicle) was developed, as shown in Figure 1. The vehicle consists of a machine vision module, a hidden surface defect detection module, and a spatial deformation module. The machine vision module is composed of high-brightness LED light sources, industrial cameras, motorized lenses, and other components. Considering the acquisition speed, the amount of stored data, and the convenience for subsequent image processing, a grayscale industrial camera was adopted. The machine vision module is capable of conducting full-section acquisition of tunnel lining images at a speed of 60 km per hour.

Using the ZJZS inspection vehicle, field data collection has been conducted in over 130 highway tunnels with different lining grades, lining types, and lane counts in Yunnan and Zhejiang provinces in China. From the collected raw images, 844 representative images containing cracks were selected.

### 2.2. Analysis of Tunnel Crack Image Characteristics

**Figure 2:**
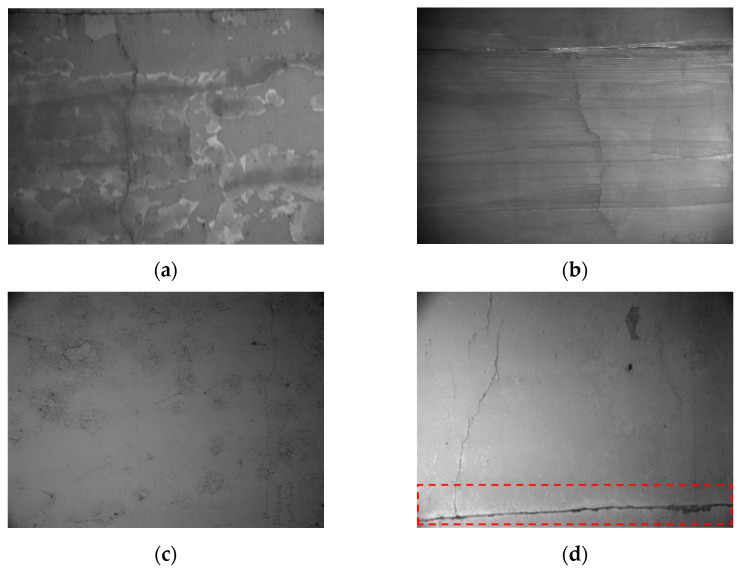
Tunnel crack images with different interference elements: (**a**) Breakage interference; (**b**) Leakage traces; (**c**) Stain interference; (**d**) Construction joint.

In order to segment the crack areas, an analysis of the characteristics of images containing crack areas was conducted. As shown in Figure 2, the collected lining crack images exhibit the following characteristics:

1. Massive image data
  The inspection vehicle captures a vast number of tunnel lining images (167 GB of data per kilometer) while in motion, resulting in a large volume of image data that requires thorough inspection;
2. Complex background with many interfering elements similar to crack features
  Image recognition and segmentation are often carried out based on feature differences; however, the breakage, leakage traces, stains, linear cobwebs, construction joints, and other interfering elements in highway tunnels are widely distributed and have complicated characteristics at different spatial locations, which interfere with recognition;
3. Diverse lining surface materials and variable image characteristics
  Highway tunnels are commonly constructed using the borehole-blasting method, resulting in in situ cast tunnel linings with variable surface roughness, decorative materials, and colors;
4. Large-size lining image with small crack proportion
  Given the large cross-sectional dimensions of highway tunnels, the speed of inspection vehicle and the frame rate limitations of the camera result in capturing a broad image range, where crack pixels occupy only an extremely small proportion less than 5%;
5. Uneven lighting in the lining images
  Although additional lighting mitigates poor tunnel illumination, inconsistencies in LED light intensity result in uneven brightness across images, causing variations in gray values at different positions.

## 3. Crack Segmentation Sample Labeling and Optimization

### 3.1. Automated Crack Segmentation

As the input of deep learning, the quantity and quality of annotations determine the effectiveness of the deep learning model. The sample set used for crack segmentation in deep learning training consists of original crack images and binary images where cracks are labeled with white (255, 255, 255) and backgrounds are labeled with black (0, 0, 0). Typically, crack pixels are manually labeled, and marked and unmarked regions are then assigned different colors through computer programming.

Taking an image of 4024 × 3036 pixels as an example, the number of crack pixels often reaches the order of magnitude of 12 million. Given the large number of sample sets required for training, manual annotation is highly labor-intensive and inefficient. For example, it usually takes an experienced annotator 2–3 min to complete the annotation of a single image. Furthermore, the manual selection of crack edges limits both the efficiency and accuracy of crack segmentation.

To address this issue, image features based on the difference in gray values between the crack and background regions, as well as the continuity of the crack propagation direction, are utilized to automate the annotation of cracks. Firstly, a pre-trained semantic segmentation model is developed using open-source crack datasets such as CrackForest and Crack500. Subsequently, this model is utilized to conduct an initial segmentation of the crack images that require annotation. Finally, the initial segmentation results are manually screened: if the segmented crack area aligns with the actual crack area, it can be used directly; otherwise, the non-crack areas erroneously identified as cracks must be removed. In cases where no crack area is present in the entire image, a line with a pixel width of 1 need to be marked artificially in the crack area.

Following this, morphological dilation operations are applied to the preliminary segmentation results, utilizing a 10 × 10 convolution kernel to compute the local maximum within each region. This operation serves to connect narrow crack discontinuities and smooth the crack contours. Using an axis extraction algorithm, the preliminary segmented crack axis is extracted. The connected domain analysis is performed on the preliminarily segmented crack axis to extract the coordinates of connected domains with an area greater than 100 pixels, thereby filtering out isolated noise points.

**Figure 3:**
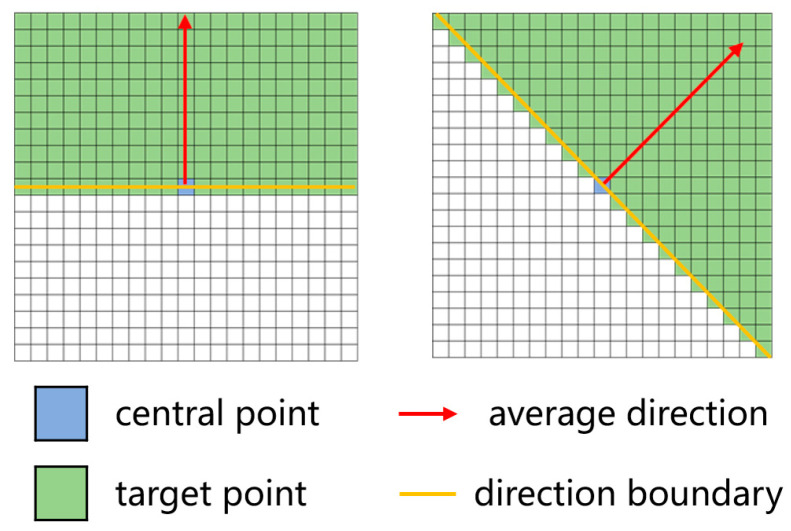
The neighborhood within ±90° of the average direction.

Using the 20 adjacent pixel coordinates close to each endpoint, the average direction (see Formula (1)) is calculated. Centered on each endpoint, the pixel with the minimum gray-scale value is then located within a 21 × 21 neighborhood spanning an angle range of ±90° from the average direction (as illustrated in Figure 3). The endpoints are connected to this minimum gray-scale value point with a 1-pixel-wide line in color (255, 255, 255). Simultaneously, the direction from the endpoint to the point with the minimum gray-scale value is taken as the new direction for calculating the average direction of subsequent points. Using this minimum gray-scale value point as the new center, the algorithm continues searching for the minimum value within a 21 × 21 neighborhood along the newly calculated average direction. If the gray-scale value difference between the identified minimum gray-scale value point and the corresponding center point is greater than 10, the algorithm stops, indicating it has been moved beyond the crack region. Furthermore, if an intersection occurs with an existing connected region or its extension, the algorithm halts, as this signals a connection to other cracks. Subsequently, this operation is repeated for all endpoints, and automatic segmentation of cracks is completed once all the endpoints have been processed.

(1) $$\bar{\lambda }=\frac{\lambda_{j,j+1}+\lambda_{j+1,j+2}+\ldots +\lambda_{j+19,j+20}}{20},\;\lambda_{i,j}=\{\begin{array}{ll} \arctan(\frac{y_{j}-y_{i}}{x_{j}-x_{i}}) & \;\;\;\;\;x_{j}-x_{i}>0\\ \arctan(\frac{y_{j}-y_{i}}{x_{j}-x_{i}})+\pi & y_{j}-y_{i}>0,x_{j}-x_{i}<0\\ \arctan(\frac{y_{j}-y_{i}}{x_{j}-x_{i}})-\pi & y_{j}-y_{i}<0,x_{j}-x_{i}<0\\ +\frac{\pi }{2} & y_{j}-y_{i}>0,x_{j}-x_{i}=0\\ -\frac{\pi }{2} & y_{j}-y_{i}<0,x_{j}-x_{i}=0 \end{array},$$

In the formula, ${\bar{\lambda }}_{j}$ represents the average direction at location j, $\lambda_{i,j}$ represents the direction between points i, and j, $x_{i},x_{j}$ separately represent the x-coordinate of point i,j, and $y_{i},y_{j}$ separately represent the y-coordinate of point i,j.

### 3.2. Optimization of Automated Crack Segmentation Results

**Figure 4:**
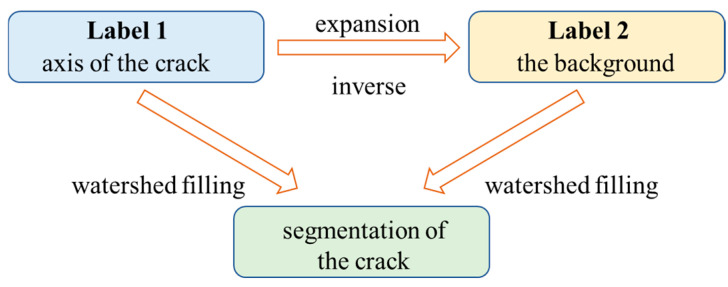
Flowchart for the watershed algorithm filling process.

To optimize the segmentation of crack edge pixels in images following automatic crack detection, a refined and standardized segmentation image optimization method that integrates axis extraction and the watershed algorithm is proposed, as illustrated in Figure 4. The process is as follows:

1. For the automatically annotated image, an axis extraction algorithm is first applied to extract the crack axis;
2. Label the crack axis as Lable1, expand the axis area by 10 pixels, and label the inverse area as Label 2;
3. Use Label 1 and Label 2 as catchment basins, and then ‘pour water’ into them until a watershed boundary formed between the two. This boundary represents the new edge of the crack, thereby achieving standardized segmentation of the crack region.

**Figure 5:**
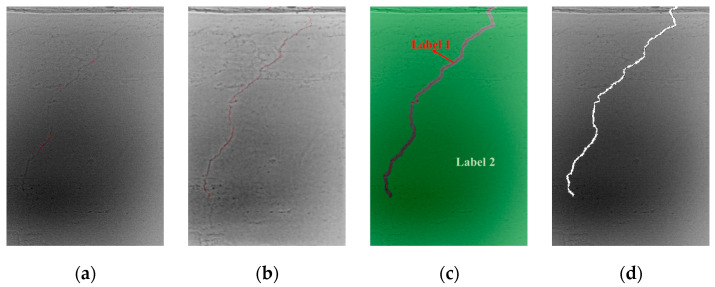
Crack image annotation and optimization: (**a**) A prepared raw images with cracks; (**b**) Automated crack segmentation results; (**c**) Segmentation result using watershed; (**d**) Standardized segmentation result of cracks.

**Figure 6:**
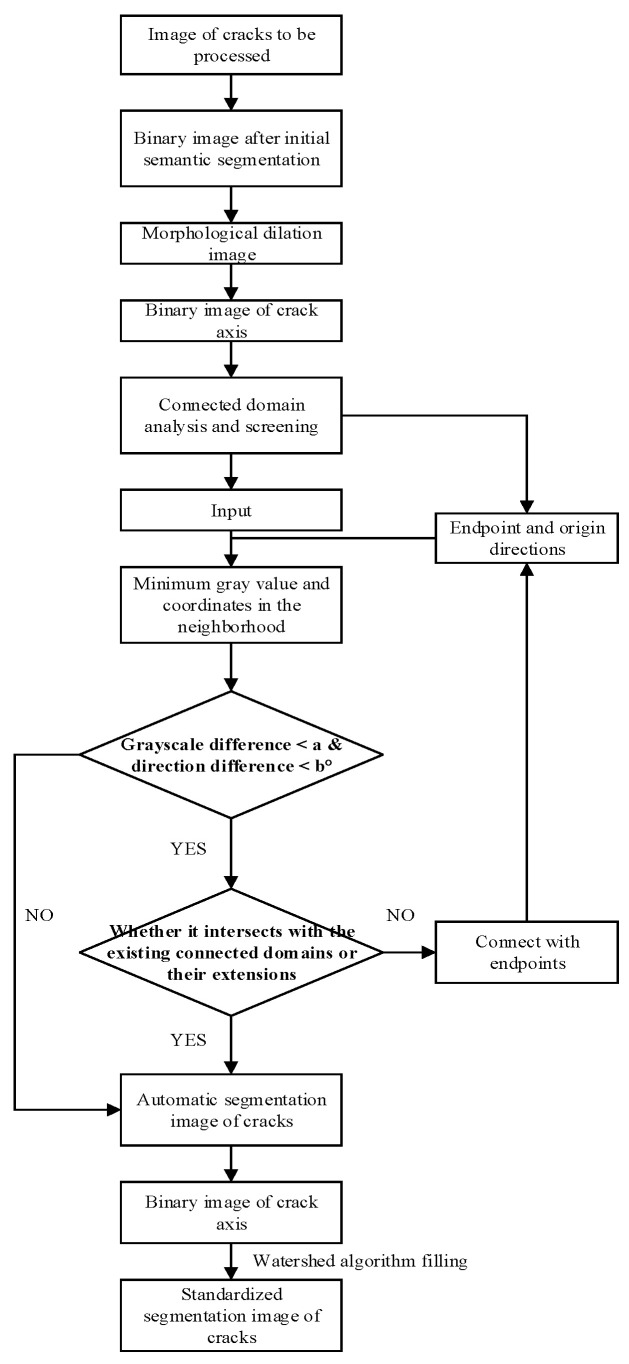
Flowchart for automatic crack segmentation.

In Figure 5a, only a small portion of the cracks in the raw image awaits annotation. After applying an automatic segmentation algorithm, the longitudinal filling of the entire length of the cracks is completed, as shown in Figure 5b. Subsequently, through axis extraction and dilation, the filled image is divided into two labels: the red crack area and the green background area in Figure 5c. The watershed algorithm is then utilized to standardize the crack edges. The final segmented crack area is displayed in Figure 5d, and the complete flowchart of the automatic crack segmentation and optimization process is provided in Figure 6.

## 4. Deep Learning-Based Crack Segmentation Algorithm

In order to accurately evaluate the safety status of the tunnel structure, it is essential not only to position and quantify tunnel lining cracks but also to obtain information on their area, width, and length. Therefore, the segmentation algorithm is required to segment the cracks in the tunnel lining image at the pixel level to derive the crack parameters based on the geometric mapping relationship.

The U-Net semantic segmentation algorithm, proposed by Olaf R [25], was initially designed for segmenting cell walls under the microscope. Due to its characteristics of rapid segmentation and compatibility with small training datasets, U-Net has been widely applied in fields such as medicine and engineering. Tunnel lining cracks share structural similarities with cell walls, both being elongated and strip-shaped structures. Therefore, this section proposes an improved U-Net semantic segmentation algorithm specifically for the semantic segmentation of cracks in tunnel linings, based on the original U-Net algorithm.

### 4.1. Network Layer Optimization

**Figure 7:**
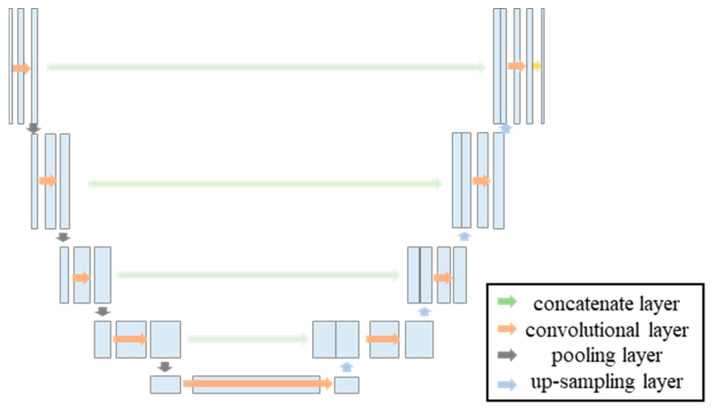
Diagram of the traditional U-Net network architecture.

The traditional U-Net algorithm network architecture, as shown in Figure 7, processes the input image through four encoding stages, each consisting of two convolutional layers and one pooling layer. This is followed by four decoding stages, each comprising one upsampling layer and two convolutional layers.

During the decoding process, each upsampled output is concatenated with the feature map of the corresponding encoding stage, preserving image details. Finally, a 1 × 1 convolutional layer is employed to reduce the dimensionality of the decoded result, and a Sigmoid activation function is used to classify each pixel.

The number of convolutions, pooling operations, and upsampling steps, which correspond to the number of layers in the model network, have an impact on image feature extraction and the final image segmentation.

**Figure 8:**
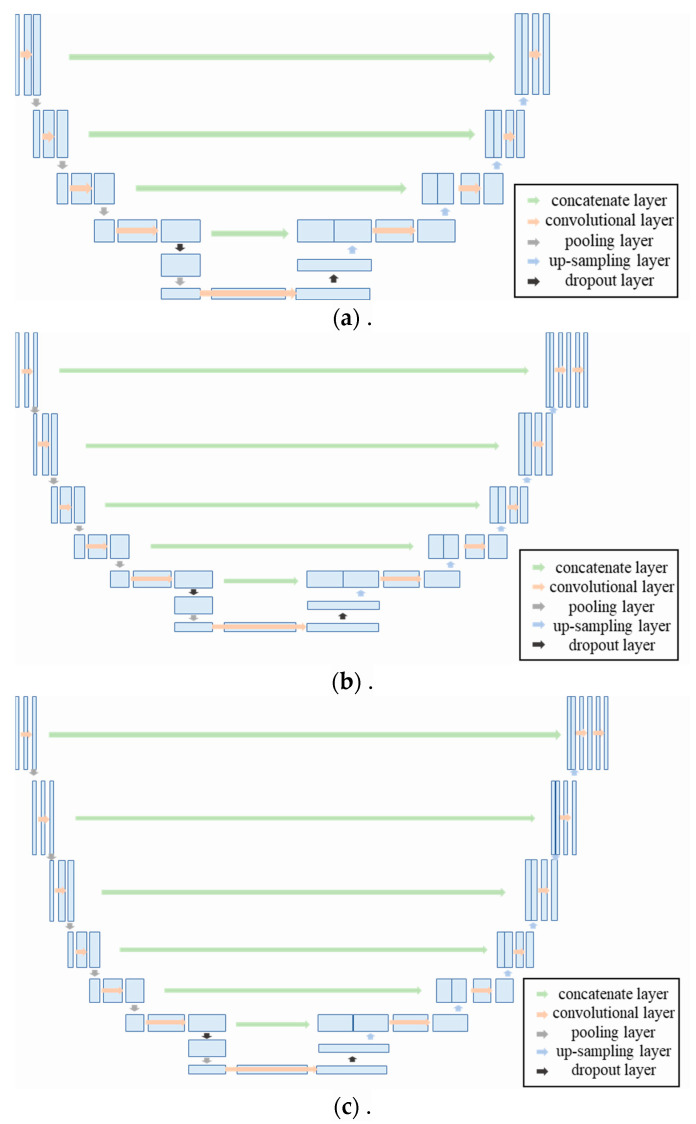
Model architectures with modified number of encoding and decoding layers: (**a**) 4-layer encoding and decoding architecture; (**b**) 5-layer encoding and decoding architecture; (**c**) 6-layer encoding and decoding architecture.

Therefore, to determine the optimal number of layers in the U-Net network for crack segmentation, the encoding and decoding stages of the traditional U-Net are modified to include 4, 5, and 6 layers, respectively. The modified network architectures are shown in Figure 8. Simultaneously, in order to prevent overfitting and enhance the model generalization capability, a dropout layer is incorporated at both the final decoding stage and the initial encoding stages. The accuracy of these three modified encoding–decoding structures is then compared through training and testing.

### 4.2. Optimization with Residual Structure

In convolutional neural networks, a model with a deeper number of layers can be achieved by adding several layers of identity mappings to a model with a shallower number of layers. Therefore, theoretically, the model with a deeper number of layers should perform better than the model with a shallower number of layers.

However, in the training process, an increase in the number of layers also increases the number of parameters and activation functions, thereby reducing the weight adjustment coefficients during the forward propagation of the loss function. Additionally, the nonlinear convolution process makes it challenging to achieve true identity mappings, resulting in a phenomenon where the model accuracy initially improves with an increase in the number of layers, but then degrades, displaying a degradation trend as the number of layers continues to grow. To address this issue, K. He [26] proposed Residual Neural Networks.

**Figure 9:**
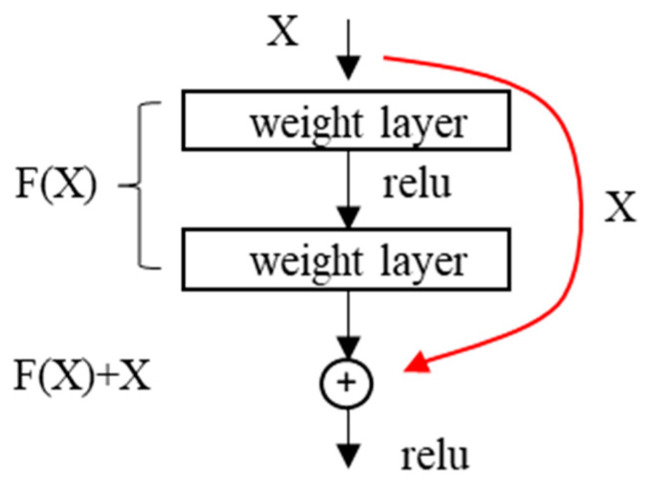
Diagram of the shortcut connection structure in ResNets.

As shown in Figure 9, Residual Neural Networks (ResNets) address the degradation issue that occurs when the increasing number of layers in a model network, by incorporating shortcut connections into Convolutional Neural Networks. These shortcut connections transform h(x) into f(x) + x, thereby converting the identity mapping (f(x) = x) into a residual structure represented by f(x) = h(x) − x. This transformation effectively changes the identity mapping problem into a zero-mapping problem, simplifying the implementation of identity mapping without increasing the number of parameters. As a result, ResNets mitigate the degradation issue associated with the addition of more layers in the network.

**Figure 10:**
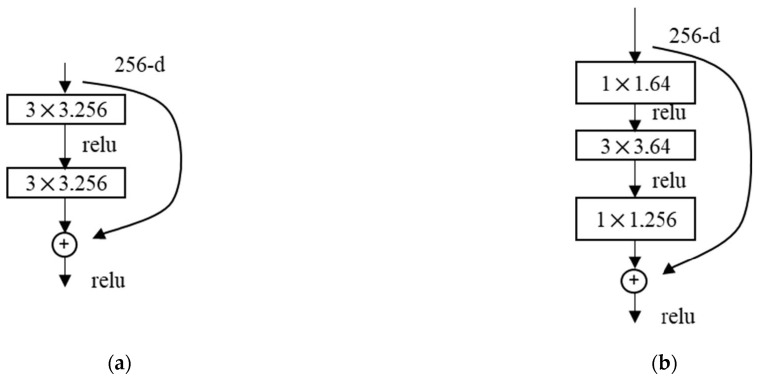
Improvement in residual block structure: (**a**) Double convolutional residual module; (**b**) Convolutional module after dimensionality reduction.

In practical applications of the shortcut connection module in ResNets, to reduce computational costs and improve efficiency, the two 3 × 3 convolutional blocks, as shown in Figure 10a, are optimized into a residual block consisting of two 1 × 1 convolutions and one 3 × 3 convolution, as depicted in Figure 10b. This optimized structure first reduces the dimensionality of the input parameters before convolving and then restores it, thereby achieving a balance between accuracy and model parameter count. For example, with a 256-dimensional input, this approach can reduce the computational load by a factor of 17. This residual structure is currently widely used in neural networks [27,28].

However, due to the unique structure of the U-Net network, the residual structure needs to be adjusted for application in the U-Net network. In ResNets, the shortcut blocks with identity mapping capabilities enable the unachievable identity mappings within Convolutional Neural Networks to be realized more efficiently and accurately. Drawing on the concept of shortcut blocks in residual structures and combining it with the characteristics of the U-Net semantic segmentation network structure, two different “Res-UNet” network architectures have been designed.

(1) Res-UNet1

**Figure 11:**
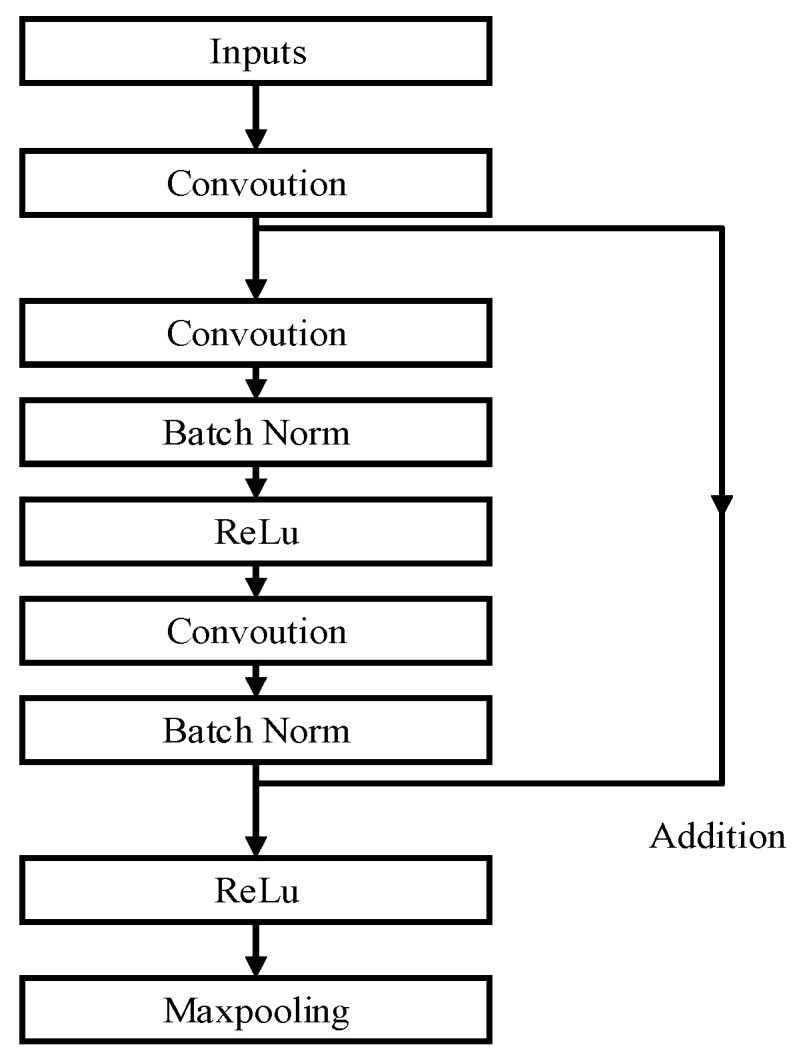
Diagram of the Rse-UNet1 residual block architecture.

In Res-UNet1, the two 3 × 3 convolutional structures at each layer during the encoding and decoding process are improved by introducing an identity mapping shortcut connection between them. Subsequently, two more 3 × 3 convolutions are performed. Prior to the activation of the final convolution, the identity mapping is fused with the convolutional regularization result before activation, thereby introducing a residual structure into the U-Net semantic segmentation network, as illustrated in Figure 11.

These improvements allow the original two 3 × 3 convolutions to flexibly vary from one to three 3 × 3 convolutions through the residual structure, which enables the model to adaptively select the most suitable parameter configuration during the training process and thus improves the model performance.

(2) Res-UNet2

Res-UNet2 integrates two 3 × 3 convolutions at each layer of the encoding and decoding processes as a unit. A shortcut connection for identity mapping is introduced before the convolutions, followed by two 3 × 3 convolution operations. Before the final convolution activation, the results of the convolution and the identity mapping are merged.

**Figure 12:**
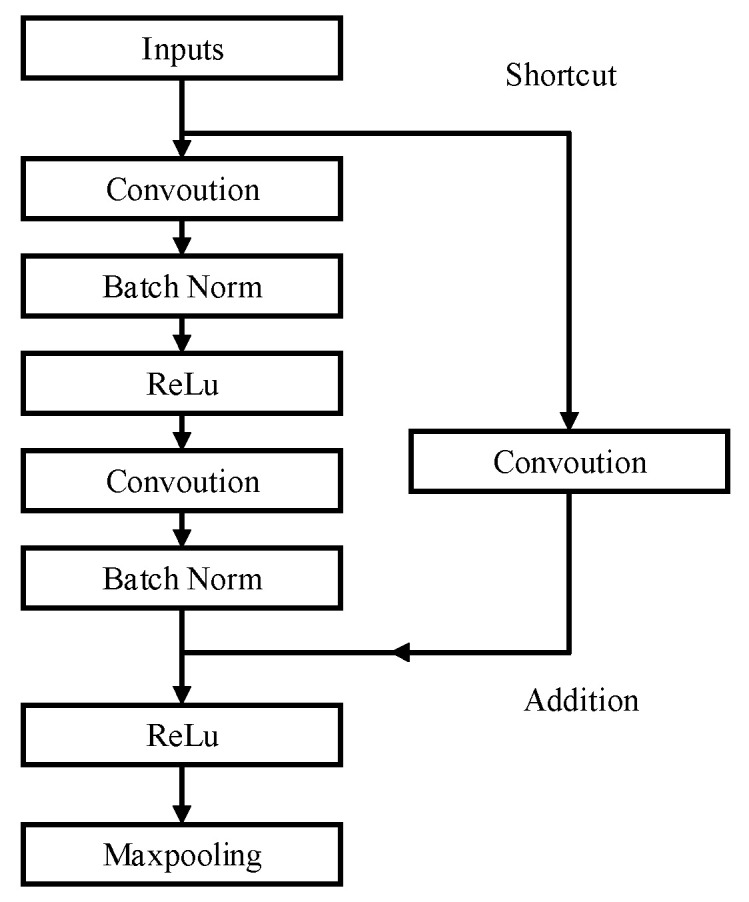
Diagram of the Rse-UNet2 residual block architecture.

Since the input of the shortcut connection is located before the first convolution, there is a difference in the number of feature map channels between the input and the output after convolution, which prevents direct mergence. To resolve this, a 1 × 1 convolution with a matching number of channels is used to adjust the number of channels in the shortcut, as shown in Figure 12. This ensures that the number of channels is consistent with the output of the convolution. Subsequently, fusion, activation, and other operations are performed.

### 4.3. Model Training

Using the crack segmentation sample annotation algorithm described in Section 3, a total of 844 crack images with typical interfering elements were annotated to establish the dataset. In order to increase the proportion of crack pixels, a crack object recognition algorithm [24] was first employed to select and crop the crack regions, thereby enhancing the crack pixel ratio. Subsequently, the dataset was divided into training, validation, and testing sets in an 8:1:1 ratio, with quantities of 676 images, 84 images, and 84 images, respectively. The server configuration parameters used for crack segmentation are shown in Table 1.

The training and analysis on the improved U-Net, Res-UNet1, and Res-UNet2 models specifically improved for tunnel lining crack segmentation were conducted to determine the optimal residual optimization structure for crack segmentation. The selected hyperparameters are presented in Table 2.

After the optimal residual neural network optimization algorithm was selected, model training was conducted using network structures with 4, 5, and 6 layers of encoding–decoding, respectively. By comparing the performance of these models on the test set, the most suitable network architecture for tunnel lining crack segmentation was determined.

## 5. Analysis of Crack Segmentation Accuracy

### 5.1. Evaluation Metrics

In semantic segmentation algorithms that classify image pixels, two commonly used evaluation metrics are pixel accuracy and Intersection over Union (IoU). Both of these evaluation metrics for semantic segmentation are based on a confusion matrix composed of true positives (TP), false positives (FP), true negatives (TN), and false negatives (FN).

(1) Pixel Accuracy

Pixel accuracy represents the proportion of correctly predicted pixels to the total number of pixels in the image. The formulas for calculating pixel accuracy and the confusion matrix are given in Formulas (2) and (3).

(2) $$\mathrm{PA}=\frac{\sum_{i=0}^{n}p_{\mathrm{ii}}}{\sum_{i=0}^{n}\sum_{j=0}^{n}p_{\mathrm{ij}}}$$

(3) $$\mathrm{PA}=\frac{\mathrm{TP}+\mathrm{TN}}{\mathrm{TP}+\mathrm{TN}+\mathrm{FP}+\mathrm{FN}}$$

where PA represents pixel accuracy, p_ij_ is the number of pixels of class i that are predicted as class j, n is the total number of pixel classes, TP stands for true positive pixels, TN stands for true negative pixels, FP stands for false positive pixels, and FN stands for false negative pixels.

(2) IOU

In the application scenarios of semantic segmentation where the number of true negatives is not a primary concern, IoU is often used to evaluate the accuracy of the model. IoU is the ratio of the intersection to the union of the prediction result and the ground truth, and its calculation formula is shown in Formulas (4) and (5).

(4) $$\mathrm{IOU}=\frac{p_{\mathrm{ii}}}{\sum_{j=0}^{n}p_{\mathrm{ij}}+\sum_{j=0}^{n}p_{\mathrm{ji}}-p_{\mathrm{ii}}}$$

(5) $$\mathrm{IOU}=\frac{\mathrm{TP}}{\mathrm{TP}+\mathrm{FP}+\mathrm{FN}}$$

Furthermore, the dice coefficient also belongs to the IoU class of evaluation metrics. It is primarily used to evaluate the similarity of segmentation results and has similarly widespread application in the field of semantic segmentation. Its calculation formula, as shown in Formula (6), is the double of the intersection between the prediction result and the ground truth divided by the sum of their absolute values.

(6) $$\mathrm{Dice}=\frac{2\times p_{\mathrm{ii}}}{\sum_{j=0}^{n}p_{\mathrm{ij}}+\sum_{j=0}^{n}p_{\mathrm{ji}}}$$

### 5.2. Comparative Analysis of Network Architectures

**Figure 13:**
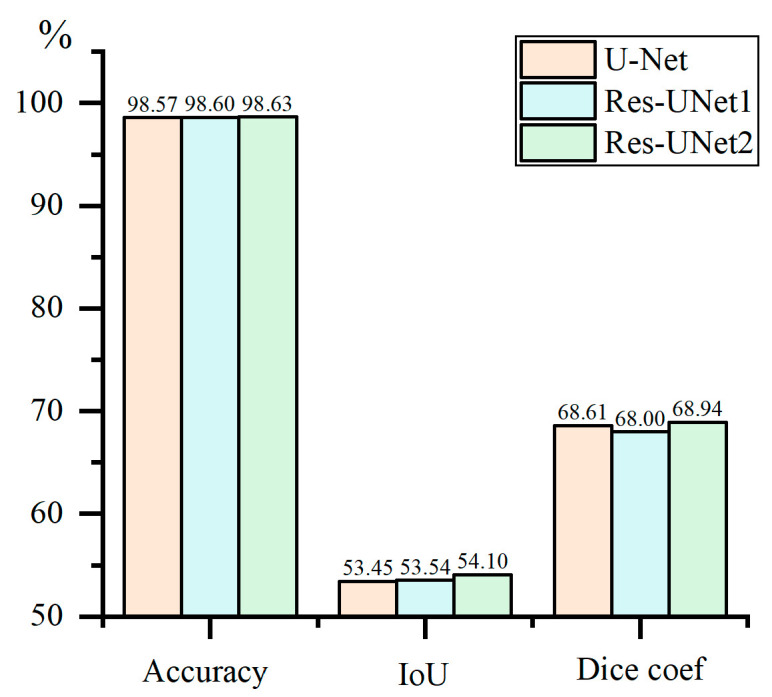
Comparison of accuracy across different network architectures.

After training with U-Net, Res-UNet1, and Res-UNet2, the test set samples are evaluated, as shown in Figure 13.

**Figure 14:**
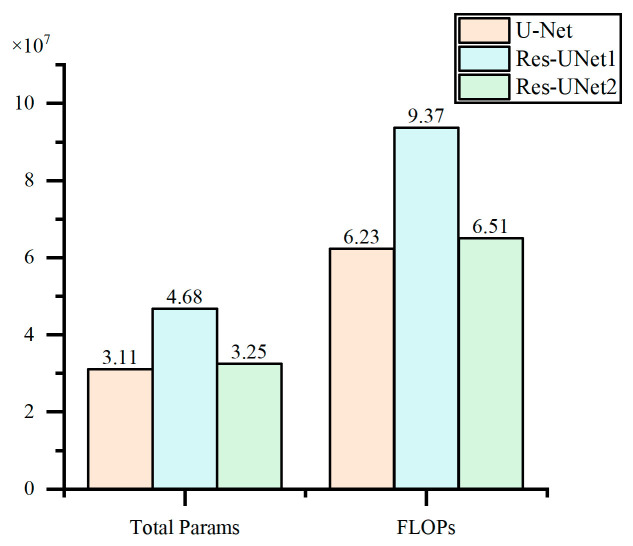
Comparison of structural complexity and computational resource consumption across various models.

Under the three evaluation metrics of Accuracy, IoU, and Dice coefficient, Res-UNet2 demonstrates significant advantages. Additionally, to compare the differences in structural complexity and computational resource consumption among different models, the total parameters and floating point operations (FLOPs) for each model were calculated, with the results shown in Figure 14. Total parameters refer to the total number of trainable parameters in a model, reflecting the scale of parameters that need to be learned and optimized during the training process. FLOPs represent the number of floating-point operations required for the model to perform one forward pass, indicating the computational demand of the model during the inference process.

The combination of U-Net and residual connections in Res-UNet2 results in a total number of parameters and FLOPs similar to the U-Net model. Moreover, it is lower than Res-UNet1, demonstrating its advantages in structural and computational complexity.

**Figure 15:**
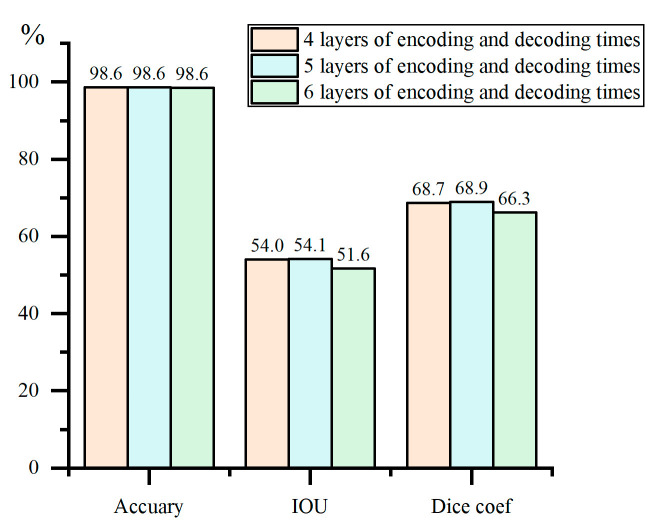
Accuracy of structures with different network layer counts.

After selecting Res-UNet2 as the most suitable model for crack segmentation through comparison, comparative training and testing of segmentation models were conducted using network structures with 4, 5, and 6 encoding and decoding layers based on Res-UNet2, respectively. The results are shown in Figure 15.

**Figure 16:**
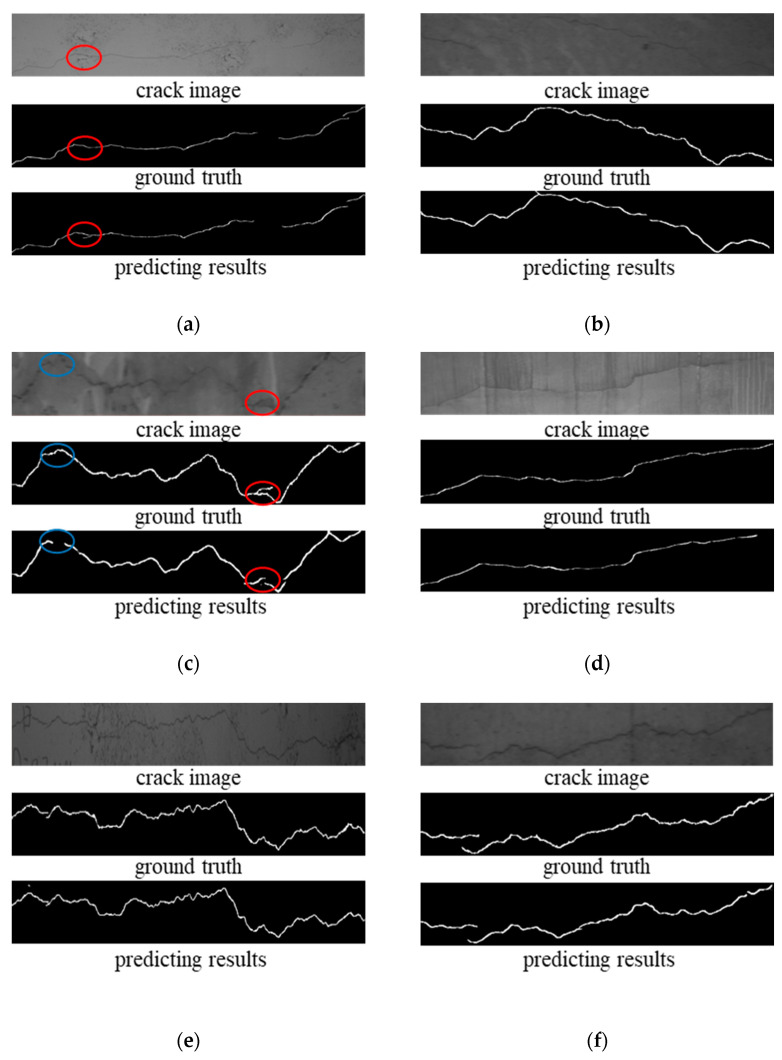
Semantic segmentation results under different interference elements: (**a**) Smooth surface; (**b**) Rough surface; (**c**) Breakage interference; (**d**) Leakage traces; (**e**) Stain interference; (**f**) Construction joint.

The model trained using the Res-UNet2 with the 5-layer encoding and decoding network structure was employed to segment typical tunnel crack images with varying surfaces and interfering elements. The results are shown in the accompanying Figure 16. The results indicate that the model can successfully segment cracks on both smooth and rough surfaces, as well as the presence of breakage, leakage traces, stains, and construction joints. However, errors are prone to occur in situations such as (a) where interfering elements overlap with crack locations (indicated by red circles), (b) where crack branching occurs (within red circles), and (c) where abrupt changes in gray values are present at crack locations (within blue circles).

### 5.3. Optimization of Crack Segmentation Results

**Figure 17:**
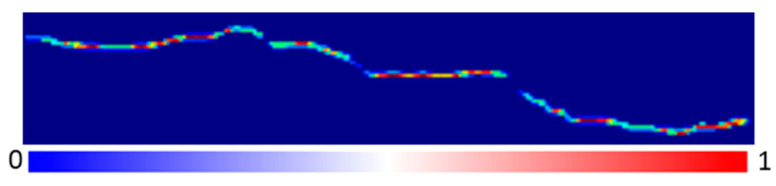
Pixel probability map for crack segmentation.

In deep learning-based semantic segmentation, the segmentation accuracy at the edges of target objects is often poor due to the transition nature of these edge pixels. As illustrated by the crack segmentation probability map in Figure 17, the transition from red to blue represents a decrease in the probability of crack pixels. It is evident that at the edges of the cracks, the probability of crack pixels decreases significantly. In practical crack semantic segmentation, most misclassified pixels are located at these edges.

**Figure 18:**
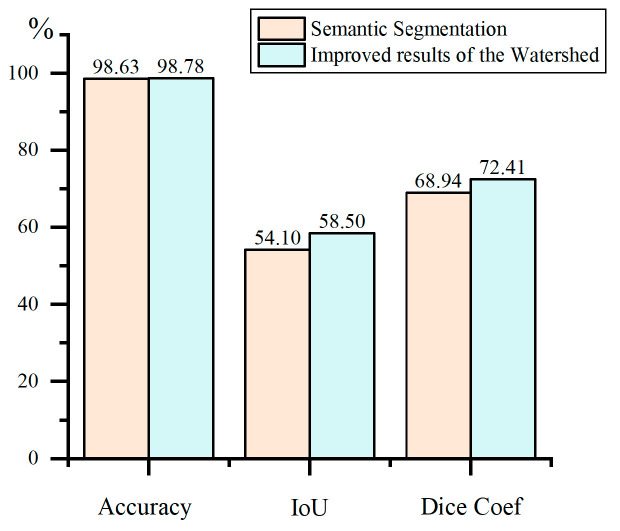
Comparison of results improved by the watershed algorithm.

To enhance crack edge segmentation, the watershed algorithm is optimized as described in Section 2.2, where the segmentation results are first extracted and then refined using the watershed algorithm. The comparison of segmentation results and evaluation metrics for typical cracks before and after using the watershed algorithm for improvement is shown in Table 3. Additionally, semantic segmentation and watershed-improved testing were conducted on all images in the test set, and the accuracy comparison under various evaluation metrics is illustrated in Figure 18. The results indicate that the improved watershed algorithm corrects misclassification at crack edges, with some improvements in metrics such as accuracy and IoU for crack segmentation.

## 6. Conclusions

In order to segment crack images with complex backgrounds in tunnels, this paper analyzes the characteristics of tunnel crack images. An automatic annotation algorithm for crack segmentation samples is then proposed, along with the introduction of a residual structure into the U-Net semantic segmentation algorithm, incorporating tailored improvements and optimizations for crack segmentation. The main findings are as follows:

1. An automatic annotation algorithm for crack segmentation samples has been established. This algorithm applies a series of morphological processing and gray-value, neighborhood, and crack propagation direction-based techniques to images that have filtrated or simple processing. By expanding the crack regions within the images, it achieves automated annotation of crack segmentation samples, reducing the workload of manual crack annotation and enhancing the efficiency of the annotation process;
2. To enhance the U-Net algorithm in deep learning, two different network architectures combining residual structures with U-Net were designed. Comparisons were also made with a structure without residual structures in terms of various accuracy evaluation metrics, model complexity, and computational complexity. Compared to Res-UNet1, the optimized structure achieved approximately a 30.5% reduction in the model and computational complexity evaluation metrics such as Total Params and FLOPs, demonstrating a significant advantage in computational efficiency. Additionally, the impact of varying the number of encoding–decoding iterations on segmentation performance was also compared. The analysis revealed that five iterations were optimal for crack segmentation, achieving an accuracy of up to 98.6% and an IoU of up to 54.1%. It achieved accurate segmentation of cracks on different lining surfaces and in various tunnel scenarios with interfering factors;
3. An optimized crack edge algorithm based on axis extraction and watershed filling has been proposed. For crack images that are automatically annotated by the automatic annotation algorithm or segmented by deep learning algorithms, morphological axis extraction, and watershed filling algorithms are applied to standardize crack edge pixels. This approach has improved accuracy to 98.78%, increased IoU to 58.50%, and raised the Dice coefficient to 72.41%. By combining the high precision of feature-based edge segmentation with the robustness of deep learning against interference, this method effectively enhances crack segmentation accuracy.

Subsequently, the database will be improved by continuously accumulating images of tunnel defects, achieving comprehensive coverage of common apparent defects within tunnels such as water leakage, spalling, tile breakage, and others. Meanwhile, by relying on the long-term accumulated tunnel image data, experimental testing of concrete, and numerical simulations, research on algorithms related to the intelligent prediction of tunnel defects will be conducted, providing support for accurate evaluation of the health status of operational highway tunnels.

## Figures and Tables

**Table 1:** The server configuration parameters.

Type	Description
Processor	Intel(R) Core(TM)i9-9940X CPU3.30 GHz 3.31 GHz,
GPU	NVIDIA GeForce RTX 2080 Ti × 4
RAM	128 G

**Table 2:** Semantic segmentation training hyperparameter table.

Type	Value
Initial learning rate	1 × 10^−4^
Batch size	20
Activation function	Sigmoid
Optimizer	Adam
Loss function	Dice coefficient

**Table 3:** Comparison table of the improved watershed method for semantic segmentation on typical crack images.

Smooth surface	Semantic Segmentation	Improved results of the Watershed
Segmentation results	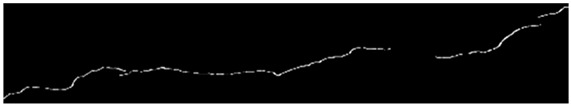	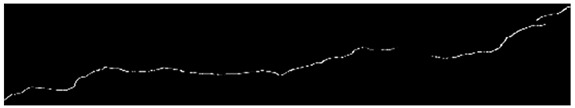
Accuracy	99.49	99.59
IoU	65.40	70.21
Dice Coef	79.08	82.50
Rough surface	Semantic Segmentation	Improved results of the Watershed
Segmentation results	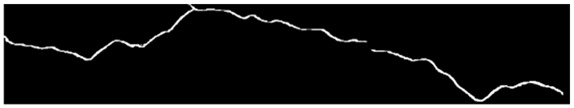	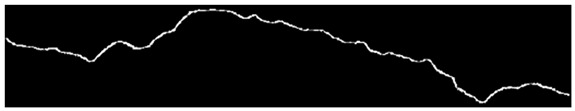
Accuracy	98.81	98.98
IoU	65.49	70.14
Dice Coef	79.15	82.45
Breakage interference	Semantic Segmentation	Improved results of the Watershed
Segmentation results	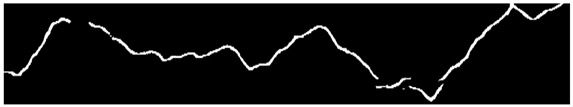	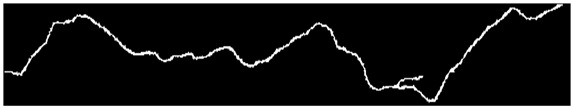
Accuracy	96.56	96.71
IoU	43.91	46.62
Dice Coef	61.02	63.60
Leakage traces	Semantic Segmentation	Improved results of the Watershed
Segmentation results	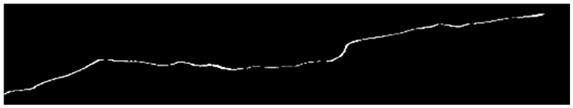	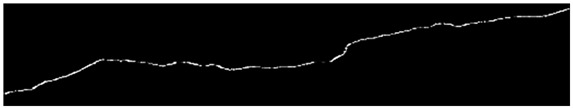
Accuracy	99.13	99.20
IoU	56.61	58.67
Dice Coef	72.30	73.95
Stain interference	Semantic Segmentation	Improved results of the Watershed
Segmentation results	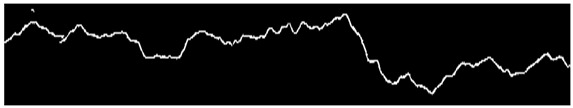	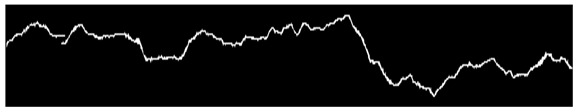
Accuracy	98.46	98.87
IoU	67.27	75.40
Dice Coef	80.43	85.97
Construction joint	Semantic Segmentation	Improved results of the Watershed
Segmentation results	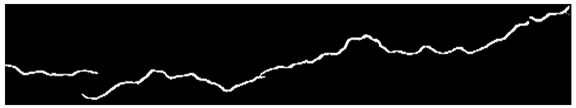	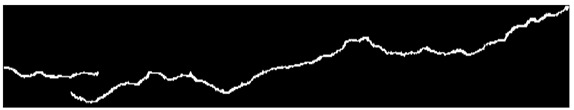
Accuracy	98.15	98.45
IoU	62.65	69.22
Dice Coef	77.04	81.81

## Data Availability

The datasets presented in this article are not readily available because the data are part of an ongoing study. Requests to access the datasets should be directed to the corresponding author.
